# Heart transplantation in patients with hypertrophic cardiomyopathy

**DOI:** 10.21542/gcsp.2018.32

**Published:** 2018-08-12

**Authors:** Marta Farrero Torres, Felix Perez-Villa

**Affiliations:** 1 IDC salud, Hospital General de Catalunya, Barcelona, Spain; 2 Universitat Internacional de Catalunya (UIC), Barcelona, Spain; 3 Hospital Clinic de Barcelona, Spain

## Introduction

Hypertrophic cardiomyopathy has a broad spectrum of clinical presentations, from asymptomatic to patients with advanced heart failure and sudden death. Treatment options are limited, especially in non-obstructive forms. A minority of patients (around 3.5%) can progress to an end-stage state, characterized by systolic dysfunction and restrictive ventricular filling, related to extensive fibrotic replacement and chamber remodeling. In these cases, life expectancy is significantly reduced: a mean 3-year survival time has been reported^1–3^.

Heart transplantation can be a life saving therapeutic option for patients with hypertrophic cardiomyopathy in a burn-out end-stage phase. Patients showing signs or symptoms of advanced heart failure should be early considered for aggressive management. Special attention must be paid to patients with ejection fraction <50% or restrictive diastolic pattern, new-onset atrial fibrillation, increased extension of left ventricular fibrosis, elevated plasma levels of natriuretic peptides or declining oxygen consumption rates^4,5^. Development of severe pulmonary hypertension is common in this population and can constitute a contraindication for heart transplantation. In other populations, it could be overcome by the use of left ventricular assist devices, but these have shown to perform poorly in restrictive physiology ventricles. The bad short-term prognosis of patients with end-stage heart failure and hypertrophic cardiomyopathy together with the specific difficulties for heart transplantation and circulatory support in this population make advisable an early consideration for aggressive therapies, as the window of opportunity may be short.

## Pulmonary hypertension

The development of pulmonary hypertension is common in left-sided cardiopathies, including valvular disease and heart failure with preserved or reduced ejection fraction. It is mainly related to the presence elevated left ventricular filling pressures due to diastolic dysfunction. In the case of hypertrophic cardiomyopathy, diastolic dysfunction, left ventricular outflow obstruction, mitral regurgitation and, in a minority of patients systolic dysfunction, can be present and cause the development of pulmonary hypertension^6–9^.

Although few groups have studied pulmonary hypertension specifically in hypertrophic cardiomyopathy population, their findings are consistent throughout the studies: pulmonary hypertension is common, and its frequency increases with the severity of the cardiomyopathy, from 20% in patients with no outflow tract obstruction to 70% in patients with end-stage disease. Moreover, the presence of pulmonary hypertension, particularly when it is associated to right ventricular dysfunction is a significant prognostic factor in this population^10^.

Irreversible pulmonary hypertension, with increased pulmonary vascular resistance can be a contraindication for heart transplantation, since it is an important cause of post-operative right ventricular failure, graft dysfunction and mortality. Pre-transplant treatment options include a pharmacological approach with pulmonary vasodilators or the implant of a left ventricular assist device^11^.

The use of pulmonary vasodilators in type 2 pulmonary hypertension is not recommended, due to consistently deleterious results in several clinical trials with prostaglandins, phosphodiesterase-5 inhibitors or endothelin antagonists. Nevertheless, its use in a highly selected pre-transplant population, allowing patients to reach candidacy has been reported in small cohorts, with good results^12^.

## Ventricular assist devices (VAD)

Ventricular assist devices are increasingly used to support patients with left ventricular dysfunction and advanced heart failure to transplant. In fact, nowadays 50% of the heart transplants performed worldwide are bridged with a VAD^13^.

Despite this fact, VAD implantation in hypertrophic cardiomyopathy is difficult, and these patients have not been enrolled in any major study. The opportunity to provide more hypertrophic cardiomyopathy patients mechanical support as a bridge to transplant has been limited by the challenges of VAD placement in this hearts, including small left ventricular chamber size (transverse end-diastolic dimension <55 mm), presence of left ventricular hypertrophy in the mid and distal left ventricular, as well as left ventricular muscle bundles, each of which could impede and obstruct the inflow cannula.

Pre VAD implantation, intra-operative myomectomy, or atrial placement of the device are surgical strategies reported in isolated cases, that may help to overcome the specific difficulties in this population. Some centers have reported their experiences with VAD implantation in restrictive and hypertrophic cardiomyopathies: compared to patients with dilated cardiomyopathies, they seem to be more susceptible to right ventricular dysfunction and hypotension, long term use of inotropes and driveline infection^14^.

That many hypertrophic cardiomyopathy patients may not qualify for VAD placement represents an impediment for irreversible pulmonary hypertension management in this population, as well as a major obstacle to achieving higher priority on the transplant list, since proposed organ allocation schemes place higher transplant priority on patients supported with VADs (over those supported with inotropic medications).

## Heart transplantation

Heart transplantation should be considered in hypertrophic cardiomyopathy patients showing refractory heart failure signs and symptoms, or considered in an end-stage state. Younger patients have a faster progression to end-stage disease in hypertrophic cardiomyopathy, so these patients with heart failure symptoms should be early recognized and carefully monitored^15^.

Heart transplant indication can be difficult in the younger population, since functional capacity is usually overestimated by clinicians. Common markers of bad prognosis are a drop in ejection fraction <50%, and the presence of a very small ventricular cavity, that is not able to fill adequately when heart rate increases.

Oxygen consumption stress tests can be performed in patients with no outflow obstruction and can provide useful information on heart performance. A decrease in oxygen consumption on exercise or peak oxygen consumption <50% of expected values adjusted for age and sex should be considered indicators of heart transplantation.

Traditional oxygen consumption threshold values for transplantation (12–14 ml/kg/min) may underestimate the risk of death in the young hypertrophic cardiomyopathy population; therefore, stress test need to be carefully interpreted and adjusted values should be preferred. Neither normal ejection fraction nor peak VO2 >14ml/kg/min should exclude drug refractory severely symptomatic HC patients from heart transplant consideration. The appearance of pulmonary hypertension on follow-up should also be considered as a red flag to start heart transplant evaluation, since it is the main cause for transplant contraindication and post-transplant mortality in this population.

Mortality on waiting list of hypertrophic cardiomyopathy patients is 10-15% and it does not differ significantly from the ischemic or non-ischemic dilated cardiomyopathy population awaiting heart transplantation. Although novel approaches, such as new allocation systems or the use of circulatory support devices, seem to have resulted in lower overall waitlist mortality, patients with hypertrophic cardiomyopathy have not benefited to the same extent from these strategies. Use of ICD and inotrope agents seem to decrease waiting list mortality in hypertrophic cardiomyopathy population^16–18^.

Post-transplant mortality in the hypertrophic cardiomyopathy group has been reported to be >90% at 1 year and >80% at 5 years, and seems to be slightly better than in other transplant populations, probably related to a younger age and less comorbidities in this group.

Overall, we could conclude that when implemented in a timely fashion, heart transplantation can represent an excellent option with good 10-year survival, supporting the view that it is the only option to restore longevity among hypertrophic cardiomyopathy patients with advanced HF.

Operative and post-operative management of heart-transplanted patients due to hypertrophic cardiomyopathy does not differ from other etiologies, and will be reviewed briefly in the following lines.

## Surgical procedure

Heart transplantation is performed with either of the following techniques:

• Shumway-Lower technique: the ventricles are excised, leaving the great vessels, and left atrium of the recipient. The donor heart is then sewn to these areas. This technique is associated to permanent sinus node dysfunction in up to 5% of cases (most of them requiring a permanent pacemaker).
• Bicaval anastomosis: The right atrium of the donor remains intact. This is most commonly used technique today.

## Immunosuppressive therapy

Immunosuppression is started soon after surgery. Several regimens can be used, usually including a calcineurin inhibitor (tacrolimus or cyclosporine), micophenolate mophetil and corticoids. At the time of transplantation, initiation of tacrolimus or cyclosporine is delayed postoperatively in patients at high risk for nephrotoxicity, and induction therapy (such as antithymocyte globulin or an IL-2 receptor antagonist such as basiliximab) may be used to permit delay or minimization of nephrotoxic calcineurin inhibitors.

## Early postoperative complications

• Right heart failure: Frequently associated to recipients with elevation of pulmonary vascular resistance.
• Biventricular failure: It may be due to poor preservation of the graft or to myocardial abnormalities present before graft harvesting.

## Rejection

• Hyperacute rejection: Catastrophic complication that may be seen in patients with preformed antibodies or in patients receiving an ABO-incompatible graft.
• Cellular Rejection: The classic form of rejection is characterized by perivascular infiltration of lymphocytes with subsequent myocyte damage and necrosis if left untreated. Myocardial biopsies are performed routinely to all heart transplant recipients, at least during the first sixth months, in order to diagnose cellular rejection.
• Antibody mediated rejection is less frequent and more difficult to characterize and diagnose. The antibody deposition into the myocardium results in global cardiac dysfunction.

## Infectious complications

Infection is common in organ transplant recipients. The types of infections expected in cardiac transplant recipients vary, depending on the time from transplantation. This is because the intensity of immunosuppression administered varies directly with the propensity for rejection, and the propensity to reject decreases over time. Bacteria and viruses account for more than 80% of infections after transplantation.

## Renal dysfunction

Immunosuppressive therapy with calcineurin inhibitors has improved both graft function and survival in heart transplantation. However, calcineurin inhibitor-induced-induced nephrotoxicity still remains a serious clinical challenge. Chronic calcineurin inhibitor nephrotoxicity is characterized by a decrease in glomerular filtration rate (GFR), afferent arteriolopathy, and striped tubulointerstitial fibrosis. About 10% of heart transplant recipients develop stage four-kidney disease (with a GFR <30 mL/min) and need kidney transplantation or renal replacement therapy because of the use of calcineurin inhibitors for immunosuppression . Close monitoring of tacrolimus and cyclosporine blood levels is critically important to limit progressive decline in renal function, because there is no known treatment for preventing or reversing nephrotoxicity.

## Coronary artery vasculopathy (CAV)

CAV was the largest problem when heart transplantation began and continues to be a major concern and focus of research. CAV is characterized by myointimal hyperplasia of small and medium-sized vessels. The lesions are diffuse and may appear any time from 3 months to several years after implantation. The inciting causes are unclear, though cytomegalovirus (CMV) infection and chronic rejection have been implicated. Coronary vasculopathy develops in 30% to 40% of heart transplant recipients within 5 years, and much over the years has not reduced the incidence. Currently, there is no treatment other than retransplantation.

## Malignancy

Following heart transplantation, malignancy is identified in 3% to 18% of the recipients, with an estimated risk of 1% to 2% per year. It ranks second to coronary vasculopathy as a major cause of mortality, accounting for 10% to 23% of all deaths following heart transplantation. Cutaneous malignancy is the most common type, seen in up to 17% of patients, with a predominance of squamous cell carcinoma. Post-transplantation lymphoproliferative disorder (PTLD) is a frequently fatal complication, occurring in 1.7% to 6% of cardiac transplant recipients.

## Outcomes

The 1-year survival rate after cardiac transplantation is as high as 84%, with a 5-year survival rate of 75% and 10-year survival rate of 59%. The functional status of the recipient after the procedure is generally excellent.
